# *Synechococcus* sp. Strain PCC 7002 Transcriptome: Acclimation to Temperature, Salinity, Oxidative Stress, and Mixotrophic Growth Conditions

**DOI:** 10.3389/fmicb.2012.00354

**Published:** 2012-10-11

**Authors:** Marcus Ludwig, Donald A. Bryant

**Affiliations:** ^1^Department of Biochemistry and Molecular Biology, The Pennsylvania State UniversityUniversity Park, PA, USA

**Keywords:** cyanobacteria, transcription profiling, RNAseq, temperature, heat shock, salinity, mixotrophy, photosynthesis

## Abstract

*Synechococcus* sp. strain PCC 7002 is a unicellular, euryhaline cyanobacterium. It is a model organism for studies of cyanobacterial metabolism and has great potential for biotechnological applications. It exhibits an exceptional tolerance of high-light irradiation and shows very rapid growth. The habitats from which this and closely related strains were isolated are subject to changes in several environmental factors, including light, nutrient supply, temperature, and salinity. In this study global transcriptome profiling via RNAseq has been used to perform a comparative and integrated study of global changes in cells grown at different temperatures, at different salinities, and under mixotrophic conditions, when a metabolizable organic carbon source was present. Furthermore, the transcriptomes were investigated for cells that were subjected to a heat shock and that were exposed to oxidative stress. Lower growth temperatures caused relatively minor changes of the transcriptome; the most prominent changes affected fatty acid desaturases. A heat shock caused severe changes of the transcriptome pattern; transcripts for genes associated with major metabolic pathways declined and those for different chaperones increased dramatically. Oxidative stress, however, left the transcript pattern almost unaffected. When grown at high salinity, *Synechococcus* sp. PCC 7002 had increased expression of genes involved in compatible solute biosynthesis and showed increased mRNA levels for several genes involved in electron transport. Transcripts of two adjacent genes dramatically increased upon growth at high salinity; the respective proteins are putatively involved in coping with oxidative stress and in triggering ion channels. Only minor changes were observed when cells were grown at low salinity or when the growth medium was supplemented with glycerol. However, the transcriptome data suggest that cells must acclimate to excess reducing equivalents when a reduced C-source is present.

## Introduction

*Synechococcus* sp. PCC 7002 (hereafter *Synechococcus* 7002) is a euryhaline, unicellular cyanobacterium, which is capable of growth over a wide range of NaCl concentrations and which is extremely tolerant of high-light irradiation (Batterton and van Baalen, 1971; Nomura et al., 2006b). Under optimal conditions [38°C, 1% (v/v) CO_2_ in air, at saturating irradiation of ∼250 μmol photons m^−2^ s^−1^], its doubling time of 2.6 h with a reduced nitrogen source is one of the fastest reported for cyanobacteria. *Synechococcus* 7002 is naturally transformable (Stevens and Porter, 1980; Frigaard et al., 2004), its genome is completely sequenced (see http://www.ncbi.nlm.nih.gov/), and a system for complementation of mutations and overproduction of proteins is available (Xu et al., 2011). These traits make *Synechococcus* 7002 an excellent platform for biotechnological applications, including the production of biofuels.

Cyanobacteria grow photolithoautotrophically, which means that light serves as the primary energy source, electrons are obtained from an inorganic source (H_2_O in the case of oxygenic phototrophs), and CO_2_ is the sole carbon source. Like all other organisms, cyanobacteria additionally require nitrogen, sulfur, and phosphorus sources, which are usually inorganic salts in the case of cyanobacteria. Cyanobacteria also require relatively large quantities of iron for growth because their photosynthetic apparatus includes numerous Fe-S proteins and cytochromes (Keren et al., 2004). Thus, global transcription studies have shown that cyanobacteria strongly regulate transcription in response to changes of irradiation and nutrient availability (Hihara et al., 2001; Gill et al., 2002; Singh et al., 2003; Wang et al., 2004; Su et al., 2006; Nodop et al., 2008; Steglich et al., 2008; Zhang et al., 2008; Ostrowski et al., 2010; Blot et al., 2011; Ludwig and Bryant, 2011, 2012; Thompson et al., 2011).

Organisms that grow autotrophically use inorganic carbon sources (CO_2_ and/or ${\text{HCO}}_{\text{3}}^{-}$). Cyanobacteria produce specialized bacterial microcompartments, carboxysomes (Yeates et al., 2008; Kinney et al., 2011), which contain ribulose 1,5-bisphosphate carboxylase/oxygenase (RuBisCO), the key enzyme for CO_2_ reduction (Tabita, 1994; Tang et al., 2011). Cyanobacterial cells also have multiple transporters for CO_2_ and bicarbonate uptake, as well as mechanisms to increase the local intracellular CO_2_ concentration within the carboxysome (Badger and Price, 2003; Yeates et al., 2008; Cannon et al., 2010). Some cyanobacteria can additionally use a few simple organic compounds, principally sugars or alcohols, as carbon and/or energy sources (Bottomley and van Baalen, 1978; Anderson and McIntosh, 1991; Eiler, 2006); *Synechococcus* 7002 can grow on glycerol as sole carbon source (Lambert and Stevens, 1986).

*Synechococcus* 7002 was isolated from a mud sample from “fish pens” on Magueyes Island, Puerto Rico (van Baalen, 1962). Closely related strains have also been isolated from sand at the edge of a clam bed in Greenwich, CT, USA (*Synechococcus* sp. PCC 7003); from a low-salinity brine pond in Port Hedland, Western Australia (*Synechococcus* sp. PCC 7117); from sea water taken on City Island, NY, USA (*Synechococcus* sp. PCC 73109); and from a lagoon near Port Gentil, Gabon (*Synechococcus* sp. PCC 8807; Rippka et al., 1979; Herdman et al., 2001). The natural habitats of these other *Synechococcus* strains are marine estuarine systems and tidal zones, in which salinity, irradiance, moisture, and nutrients can fluctuate dramatically. Organisms living in these habitats must have the ability to acclimate rapidly to variations in these and other physico-chemical parameters. Temperatures in tidal zones can increase rapidly and vary significantly, and therefore the tolerance to elevated temperature and/or the ability to survive high temperatures is another important trait for *Synechococcus* strains from similar habitats. Several global transcriptome studies have been performed for cyanobacteria, mainly in *Synechocystis* sp. PCC 6803 and *Anabaena* sp. PCC 7120, to investigate the effects of salt stress (Kanesaki et al., 2002; Postier et al., 2003; Marin et al., 2004), low temperature (Suzuki et al., 2001; Mikami et al., 2002; Ehira et al., 2005), heat shock (Suzuki et al., 2005, 2006), oxidative stress (Kobayashi et al., 2004; Li et al., 2004), and photomixotrophic growth with carbon sources in addition to CO_2_ and bicarbonate (Kahlon et al., 2006).

As part of an effort to produce a comprehensive and integrated global transcriptome database for the model cyanobacterium *Synechococcus* 7002, we present here global transcription profiles for cells grown at different salinities (3 mM NaCl, 300 mM NaCl, and 1.5 M NaCl) and at different temperatures (22, 30, and 38°C). Because *Synechococcus* 7002 is unable to grow exponentially with nitrate as nitrogen source at temperatures below 22°C, temperatures lower than 22°C were not included in this study (Sakamoto and Bryant, 1998; Sakamoto et al., 1999). We also investigated the impact of heat shock and oxidative stress on the transcriptome of *Synechococcus* 7002. Finally, we compared the transcriptome of cells grown photolithoautotrophically with CO_2_/bicarbonate with that for cells grown photomixotrophically with CO_2_/bicarbonate and glycerol.

## Materials and Methods

### Sample preparation

*Synechococcus* 7002 cultures were grown in tubes containing medium A (25-ml) supplemented with 1 mg NaNO_3_ ml^-1^ (designated as medium A^+^; Stevens and Porter, 1980; Ludwig and Bryant, 2011). Growth was monitored using a spectrophotometer (Genesys 10, ThermoSpectronic, Rochester, NY, USA) at 730 nm. The cultures were grown at 38°C with continuous illumination at 250 μmol photons m^-2^ s^-1^ and were sparged with 1% (v/v) CO_2_ in air. These optimal growth conditions are defined as “standard conditions.” Reference cells were grown under standard conditions to an OD_730 nm_ = 0.7 (Ludwig and Bryant, 2011). Growth at lower temperatures was performed at 22 or 30°C, respectively, under otherwise identical conditions. A culture was subjected to a 1-h heat shock at 47°C after cells were grown to OD_730 nm_ = 0.7 under standard conditions. In order to induce oxidative stress, methyl viologen was added to *Synechococcus* 7002 culture at OD_730 nm_ = 0.7 to produce a final concentration of 5 μM. The cells were incubated for an additional 30 min under standard conditions prior to harvesting the cells.

For cultures grown at different salinity, NaCl and KCl concentrations were both modified but other media components were unmodified. Compared to 300 mM NaCl and 8 mM KCl in standard A^+^ medium, the low-salinity medium contained only 3 mM NaCl and 0.08 mM KCl, whereas the higher salinity medium contained 1.5 M NaCl and 40 mM KCl. For mixotrophic growth of cells, standard A^+^ medium was supplemented with 10 mM glycerol. Unless specified otherwise, cultures were grown at standard light, temperature, and sparging conditions.

### RNA extraction, cDNA library construction, and SOLiD^™^ sequencing

Cells from cultures grown to a final OD_730 nm_ of 0.7 were rapidly centrifuged (5 min, 5000 × *g*, 4°C), and the cell pellets were frozen in liquid nitrogen and stored at −80°C until required. RNA samples for subsequent cDNA library construction were prepared from frozen cell pellets resulting from 20 to 30 ml liquid cultures as described previously (Ludwig and Bryant, 2011). RNA concentrations were determined with a NanoDrop ND-1000 Spectrophotometer (Thermo Scientific), and both RNA and DNA concentrations were separately determined with a Qubit System (Invitrogen).

Construction of cDNA libraries and SOLiD^™^ (Applied Biosystems) sequencing was performed in the Genomics Core Facility in the Huck Institutes for the Life Sciences at The Pennsylvania State University (University Park, PA, USA). The cDNA libraries were constructed from 0.5 μg RNA using SOLiD^™^ Whole Transcriptome Analysis Kit and were barcoded by using the SOLiD^™^ Transcriptome Multiplexing Kit. The SOLiD^™^ ePCR Kit and SOLiD^™^ Bead Enrichment Kit were used to process samples for sequencing, which was performed using either the SOLiD^™^ 3, 3 Plus, or SOLiD^™^ 4 protocols as described by the manufacturer (Applied Biosystems).

The sequence data have been submitted to the NCBI Sequence Read Archive (SRA) under accession number SRP013965.

### Data analyses

The cDNA sequence data were mapped against the *Synechococcus* 7002 genome and processed as described previously (Ludwig and Bryant, 2011, 2012). This resulted in a list of the relative transcript abundances for all open reading frames (ORFs), which is the number of sequences mapping to a given ORF divided by the total number of sequences mapping within any protein-coding region. The relative transcript abundances for two different samples or conditions were compared for all ORFs as the relative transcript abundance under the test condition divided by the relative transcript abundance under the reference condition. Data sets obtained with different SOLiD^™^ sequencing chemistries were analyzed and compared separately. For the SOLiD^™^ 3 data sets presented here, the reference data for standard growth conditions were obtained in a previous study (Ludwig and Bryant, 2011). SOLiD^™^ 4 data sets were compared to three cDNA libraries that were independently prepared with RNA derived from control cells grown under standard conditions and sequenced with SOLiD^™^ 4 chemistry. Statistical analyses were performed as previously described (Ludwig and Bryant, 2011).

## Results and Discussion

### Global transcriptome data sets for different growth conditions and perturbations

The global transcriptome of the cyanobacterium *Synechococcus* 7002 was determined for cells grown at different temperatures and salinities, after heat shock, and after induction of oxidative stress by addition of methyl viologen. Furthermore, the transcriptome was obtained for a culture that was grown photomixotrophically with glycerol. The doubling times for cultures that were continuously grown under the same conditions were compared to that of cultures grown under standard conditions (see Table 1). Approximately 4–30 million sequences were obtained for each sample, and these were mapped to the *Synechococcus* 7002 genome (Table 1). These numbers varied as a function of two principal factors: (1) the number of samples that were barcoded and pooled for one sequencing run, which determines the resolution (sequencing depth) for a given sample; and (2) the particular SOLiD chemistry that was employed for a given sample (see Text S1 in Supplementary Material). Sequences that mapped to protein-coding ORFs were further analyzed, and the calculated relative transcript levels for all genes and for all samples discussed here are shown in Table S1 in Supplementary Material. Comparisons between different conditions were performed on a gene-by-gene basis; and the results for all of those comparisons, including tests for statistical significance, are presented in Table S2 in Supplementary Material.

**Table 1:** **Number of sequences obtained by SOLiD^™^ sequencing for the samples analyzed in this study**.

Sample	Mapped reads	Mapped in rDNA regions	Percent rDNA	Remaining mapped reads	Uniquely mapped reads	Percent unique reads	Doubling time (% of standard conditions)
Standard 1^#3^	18,238,746	13,193,499	72.3*	5,045,247	4,886,185	96.8	
Standard 2^#3^	29,450,401	27,109,346	92.1	2,341,055	2,270,856	97.0	100
Standard 3^#3^	25,082,458	22,558,727	89.9	2,523,731	2,455,018	97.3	
Mixotrophic^#3^	10,439,860	9,412,386	90.2*	1,027,474	996,440	97.0	84
Oxidative stress (MV)^#3^	32,460,233	30,484,090	93.9	1,976,143	1,918,914	97.1	n.d.^$^
22°C^#3^	15,029,141	13,885,066	92.4	1,144,075	1,116,520	97.6	194
30°C^#3^	12,172,383	11,276,145	92.6	896,238	871,777	97.3	109
Standard 4^#4^	3,740,709	3,061,465	81.8	679,243	660,292	97.2	
Standard 5^#4^	5,817,054	4,638,642	79.7	1,178,404	1,140,157	96.8	100
Standard 6^#4^	5,161,758	3,704,426	71.7	1,455,423	1,413,735	97.1	
Low salt^#4^	6,423,270	5,192,513	80.7	1,222,976	1,162,636	95.1	98
High salt^#4^	5,758,000	4,755,907	82.6	999,930	972,762	97.3	270
Heat shock^#4^	6,513,432	5,788,273	88.8	720,347	699,012	97.0	n.d.^$^

*The number of reads obtained for the different samples, number of mapped reads, number of reads mapping within the rDNA regions and outside rDNA regions, and the number of reads mapping uniquely (outside rDNA regions) are given for the individual samples. Furthermore, the doubling time for cultures grown continuously under the respective conditions is given relative to that for cultures grown under standard conditions. Samples standard 1, 2, and 3 are the same as in our previous study (Ludwig and Bryant, 2011)*.

*^#3^Data sets were generated using SOLiD^TM^ 3 or 3 plus chemistry*.

*^#4^Data sets were generated using SOLiD^TM^ 4 chemistry*.

*These samples were treated to deplete rRNAs (see Ludwig and Bryant, 2011).

*^$^Short-term incubations (treatment with methyl viologen for 30 min and a 1-h heat shock at 47°C) followed growth under standard conditions*.

### Impact of growth temperature on the transcriptome

**Figure 1:**
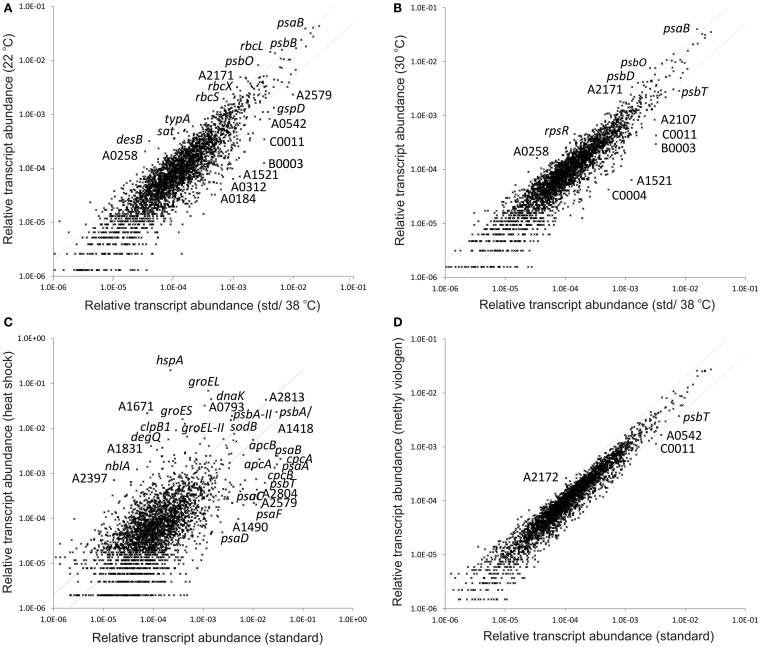
**Changes in the relative transcript abundance when cultures were grown at different temperatures, upon heat shock, and after exposure to oxidative stress**. The scatter plots show the relative transcript abundances of cultures grown at **(A)** 22°C and **(B)** 30°C compared to that for standard growth conditions at 38°C. Scatter plots shows the relative transcript abundance of a culture **(C)** exposed to a 1-h heat shock at 47°C and **(D)** incubated with 5 μM methyl viologen to induce oxidative stress after standard growth compared to that obtained for standard growth conditions. The values for the standard conditions were calculated as the mean for three biological replicates. The gray lines give twofold changes in either direction. Selected genes are identified by name/locus tag number.

Compared to other data sets presented in previous studies (Ludwig and Bryant, 2011, 2012), which included nutrient limitation and dark oxic or anoxic incubation, the changes of the global transcriptome of *Synechococcus* 7002 were relatively minor when cultures were grown at different temperatures (at least in the range from 22 to 38°C). The differences for cells grown at 22°C (Figure 1A) were somewhat greater than for cells grown at 30°C (Figure 1B), perhaps because 22°C is the lowest temperature at which cells can grow exponentially on nitrate as N-source and because cells at 22°C grew slower than cells at 30 and 38°C; the doubling time was almost twice as long compared to cells grown under standard conditions at 38°C (Table 1).

Compared to standard conditions, the maximum increase for any gene in cells grown at 22°C was an eightfold change (see Figure 1A). The *desB* gene and the genes involved in CO_2_ uptake and fixation are among those exhibiting the largest increases (see below). The largest decrease in any transcript level was about 50-fold; however, for most of the genes showing more than a ∼10-fold decrease in transcript levels, the *p*-values for the respective comparisons were relatively high, and therefore an interpretation as differential expression was not strongly supported statistically. Furthermore, many of the genes in this category are annotated as hypothetical genes. The differences between the transcriptomes of cells grown at 30 and 38°C were less pronounced; the largest increase was about fivefold and the maximum decrease was about 20-fold. At both 22 and 30°C the transcript levels for SYNPCC7002_A1521, annotated as GTPase ObgE, was ∼20-fold lower compared to cells grown under standard conditions. Transcripts for *hspA* (encoding a small heat-shock protein) were much lower in cells at 22 and 30°C (10- and 6-fold, respectively). Compared to cells grown under standard conditions, transcript levels of genes coding for chaperones were generally less abundant in the 22 and 30°C samples (Table S3 in Supplementary Material). This trend suggests that cells have a lower requirement for molecular chaperones to ensure proper protein folding at lower temperatures.

**Figure 2:**
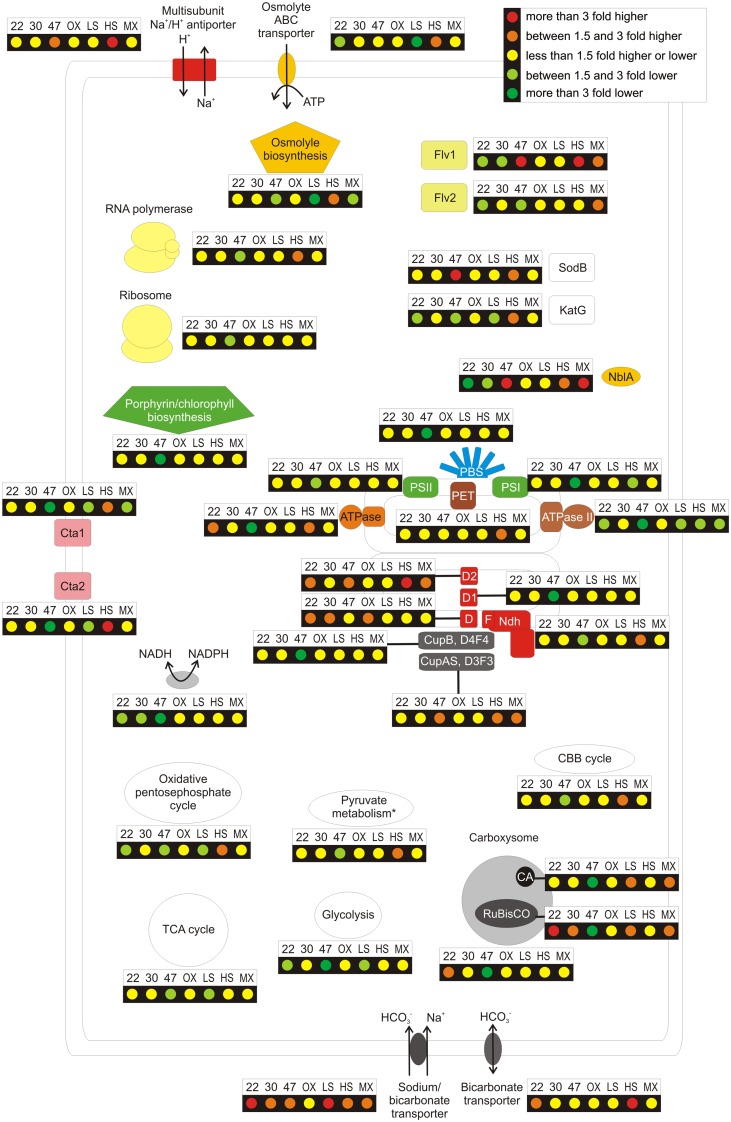
**Overview over the regulation pattern of cells in response growth at different temperatures, at different salinity, after a heat shock, when exposed to oxidative stress and when grown mixotrophically**. The figure summarizes the general regulation patterns when cultures were grown at lower temperatures (22 or 30°C) instead of 38°C (22 and 30, respectively), after a 1-h heat shock at 47°C (47), at lower (LS) or higher (HS) salinity than standard A^+^ medium, after exposure to oxidative stress (OX), which was induced by incubating the culture with 5 μM methyl viologen for 30 min in light, and for photomixotrophic growth with 10 mM glycerol supplied as organic carbon source (MX). The changes of the relative mRNA levels in cultures grown under different conditions or upon additional incubation are compared to standard photoautotrophic growth conditions. These ratios are displayed for genes coding for a selection of cellular functions, among these the photosystems (PSI and PSII), phycobilisomes, the porphyrin/chlorophyll biosynthesis, the phycobilisome degradation protein NblA, other components of the photosynthetic electron transport chain, the carboxysome, bicarbonate transporters, the NADH dehydrogenase complex, the F_0_F_1_-Ztype ATP synthase I, ATPase II (N-ATPase), and terminal oxidases. Furthermore, genes coding for the transhydrogenase, for the multisubunit Na^+^/H^+^ antiporter, for enzymes involved in osmolyte biosynthesis and for the osmolyte ABC-transporter are depicted. In addition, genes are shown for the major carbon pathways: the Calvin–Benson–Bassham cycle, glycolysis, pyruvate degradation (via pyruvate kinase and pyruvate dehydrogenase), the oxidative pentose phosphate cycle, and the tricarboxylic acid cycle. Genes coding for the RNA polymerase (core complex) and for ribosomal proteins are also highlighted; finally, the changes in the transcript levels of two flavoproteins, superoxide dismutase, and catalase are given.

An overview for changes in transcript levels for genes encoding major structural components, biosynthetic machinery, and major biochemical pathways are shown in the cellular representation in Figure 2. Only minor changes in transcript levels were observed for genes encoding components of the photosynthetic apparatus (phycobilisomes, photosystems, and photosynthetic electron transport chain), heme and chlorophyll biosynthesis enzymes, RNA polymerase, and ribosomal proteins. The *nblA* transcript level was lower in cells grown at lower temperature (Figure 2). NblA causes phycobiliproteins to become sensitive to proteolytic degradation (Baier et al., 2004; Karradt et al., 2008), and this gene is usually transcribed heavily under unfavorable conditions to promote the release of reduced carbon and nitrogen (Collier and Grossman, 1992). Transcript levels for genes encoding enzymes involved in carbohydrate degradation (glycolysis and oxidative pentose phosphate pathway) also decreased slightly (Figure 2; Table S3 in Supplementary Material). Interestingly, genes coding for the transhydrogenase (*pntA*, *pntB*, and *pntC*) also had lower mRNA levels at low temperature.

Transcript levels for genes involved in CO_2_ fixation increased at low temperature. The *rbcL* and *rbcS* genes coding for RuBisCO had approximately threefold higher transcript levels in cells grown at 22°C, but these transcripts were only ∼1.5- to 2-fold higher in cells grown at 30°C (Figure 2). Transcripts for genes encoding the structural components of the carboxysome and the gene coding for the carbonic anhydrase increased only slightly in cells grown at lower temperatures (approximately twofold at maximum). Transcripts for the *sbtA* gene, coding for a Na^+^-dependent bicarbonate transporter, increased approximately threefold in cells grown at 22°C and approximately twofold in cells grown at 30°C. Slight increases in mRNA levels were also observed for another bicarbonate transporter (*bicA*) and for genes encoding for the so-called inducible CO_2_ transporter (see Table S3 in Supplementary Material), which is a variant form of the NADH dehydrogenase complex (Ogawa and Mi, 2007; Battchikova et al., 2011). Collectively, these data suggest that cultures grown at lower temperature experience slight CO_2_ limitation. Enzymatic reactions should occur approximately threefold slower at 22°C than at 38°C (the *Q*_10_ rule). Because CO_2_ should actually be more soluble at lower temperatures, the data suggest that *Synechococcus* 7002 cells carefully regulate the production of the CO_2_ concentrating mechanism (CCM) and do not produce the CCM in excess; however, cells can adjust the amount of the CCM in response to the demand for CO_2_ to act as an electron sink. The *flv1* and *flv2* genes, encoding flavoproteins that had strongly increased transcript levels under prolonged CO_2_ limitation (Ludwig and Bryant, 2012), had lower mRNA levels in cells grown at lower temperature (twofold at maximum). This suggests that these genes are regulated in response to a different signal than the genes for CCM. Transcripts for genes encoding the F_0_F_1_-type ATPase increased, especially in the culture grown at 22°C (threefold at maximum), whereas transcripts of genes encoding a putative Na^+^-transporting ATPase (Dibrova et al., 2010) decreased.

A major acclimation process to changing temperatures in bacteria involves changes in the desaturation level of their membrane lipids (Aguilar and De Mendoza, 2006). Several fatty acid desaturases perform oxidation of fatty acids at different positions. The genome of *Synechococcus* 7002 encodes five fatty acid desaturases: *desA* (Δ12 acyl-lipid desaturase), *desB* [ω-3 (Δ15) acyl-lipid desaturase], *desC* (Δ9 acyl-lipid desaturase), *desE* (similar to Δ9 acyl-lipid desaturase), and *desF* (putative *syn*-2, Δ9 acyl-lipid fatty acid desaturase). A previous study on transcript levels of *desA*, *desB*, and *desC* in *Synechococcus* 7002 showed that *desB* transcripts were highly abundant in cells grown at 22°C, whereas *desB* mRNA was below the detection limit in a culture grown at 38°C. Transcripts for *desA* were detected in both samples, but were three times more abundant in cells grown at 22°C compared to cells grown at 38°C. Transcripts for *desC*, however, had similar levels at both temperatures (Sakamoto and Bryant, 1997). These results obtained via Northern blot hybridization agree perfectly with the data obtained in the present study using cDNA sequencing. Compared to standard conditions at 38°C, the mRNA level of *desA* increased by 2.5-fold in a culture grown at 30°C and by 2.8-fold in a culture grown at 22°C (Table S3 in Supplementary Material). The *desB* transcripts increased by almost threefold in cells grown at 30°C and eightfold in cells grown at 22°C. Interestingly, the relative transcript abundance of *desB* was easily detectable by cDNA sequencing and was only about fourfold lower than that for *desA* under standard growth conditions (see Table S1 in Supplementary Material). The observation that *desB* transcripts are observed at elevated temperatures but increase dramatically at 22°C are consistent with the previous observations made for *Synechococcus* 7002 and *Synechocystis* sp. PCC 6803 (Los et al., 1997; Sakamoto and Bryant, 1997). However, these observations are not consistent with the idea that the transcription of desaturase gene(s) is controlled by a transcription factor. This pattern is more consistent with the previous suggestion that *des* transcripts, especially those for *desB*, are inherently more stable at lower temperature (Sakamoto and Bryant, 1997). Temperature-dependent changes of the mRNA secondary structure have been described as control elements for ribosome binding and initiation of translation, and these RNA elements were subsequently named RNA thermometers (Kortmann and Narberhaus, 2012). Based on similar mechanisms, a stabilization of transcripts could be achieved at lower temperatures, which would result in an accumulation of those mRNAs. At higher temperatures the secondary structure of the same mRNAs could change, which would in turn result in quick degradation, and consequently, low levels of the respective transcripts would be observed at higher temperatures.

As found by Northern blot-hybridization analysis, *desC* transcript levels were similar in cells grown continuously at 22, 30, and 38°C. Transcript levels for *desE* increased approximately twofold in cells grown at 22 and 30°C compared to cells grown at 38°C, whereas the *desF* transcript level was unaffected by growth temperature (*p*-values did not support a significant difference). In a previous transcriptome study in *Synechococcus* 7002, it was reported that *desF* is part of an operon of four genes induced by low O_2_ (Ludwig and Bryant, 2011). Increased transcript levels of three fatty acid desaturase genes (*desA*, *desB*, and *desC*) have also been reported for *Nostoc* sp. PCC 7120 when cultures were incubated at lower temperatures for 2 h at the same light level (Ehira et al., 2005). The same study also reported that transcript levels for nearly 300 genes either increased or decreased after a 2-h incubation at lower temperature. However, the transcript pattern after cells were incubated at lower temperature for 30 min was reported to be somewhat different, which suggested that many of the changes were transient (Ehira et al., 2005).

### Exceeding the optimum growth temperature: The heat-shock response as seen by transcriptomics

The optimum growth temperature for *Synechococcus* 7002 is 38°C (van Baalen, 1962), and this temperature has been used for the growth of all cells under standard conditions (Ludwig and Bryant, 2011, 2012). Temperatures substantially higher than the optimum cause a response known as heat shock, under which cell growth is much slower or completely inhibited, and mechanisms are induced to allow survival under these unfavorable conditions (Guisbert et al., 2008; Rasouly and Ron, 2009). To induce a heat-shock response in *Synechococcus* 7002, a culture that had been grown under standard conditions was incubated at 47°C for 1 h – a treatment that almost immediately stops cell growth, but from which the culture can recover when incubated at 38°C again, which was determined in a separate experiment. A comparison of the transcriptome obtained for cells that had been subjected to a heat shock relative to standard conditions is shown as a scatter plot in Figure 1C.

The changes of the global transcriptome observed for the sample subjected to heat shock were easily the most extensive observed for any of the conditions tested in this study and in our previous global transcriptome studies in *Synechococcus* 7002 (Ludwig and Bryant, 2011, 2012). Most of the genes showing the highest increases in mRNA levels were genes encoding different chaperones (e.g., *hspA*, *groEL*, *groES*, *groEL-II*, and *dnaK*). The ∼900-fold increase of *hspA* transcripts was the largest increase observed in the cells subjected to heat shock; *hspA* transcripts accounted for nearly 20% of the total mRNA in cells after heat shock. When cultures were grown at lower temperatures (30 and 22°C, see above), transcripts for *hspA* were much less abundant compared to standard growth at 38°C (5- to 10-fold, respectively), which indicated a clear trend: *hspA* transcript levels increased directly as the temperature increased. After a 1-h heat-shock treatment, transcript levels for other chaperones increased 30- to 50-fold (Table S3 in Supplementary Material). Although this is a smaller effect than observed for *hspA*, it still represented a major change in transcript abundance compared to changes for cells that were subjected to other physico-chemical stresses. Remarkably, several of the genes that are annotated as DnaJ and DnaK chaperones did not show increased transcript levels after a 1-h heat shock (Table S3 in Supplementary Material). Similar transcription changes for chaperones have also been observed in *Synechocystis* sp. PCC 6803, both at the mRNA level and at the protein level (Suzuki et al., 2006). In *Synechococcus* sp. PCC 7942 HspA has been suggested to protect phycobiliproteins from degradation under heat stress as well as oxidative stress (Nakamoto et al., 2000; Nakamoto and Honma, 2006). The two GroEL homologs present in cyanobacteria have been studied in *Thermosynechococcus elongatus* BP-1 (Sato et al., 2008). GroEL-2 was not essential under optimum growth conditions, but played important roles during acclimation to heat shock and low temperature. This trend may not occur universally among cyanobacteria, because transcript levels of *groEL-2* were only about threefold lower compared to *groEL* under standard conditions in *Synechococcus* 7002 (Table S1 in Supplementary Material).

Transcripts of genes coding for the major metabolic functions, i.e., photosynthesis and CO_2_ fixation, were strongly affected by heat shock. The mRNAs of most genes encoding subunits of PS I, PS II, phycobiliproteins, F_0_F_1_-ATPase, RuBisCO, CCM, and for the entire Calvin–Benson–Bassham (CBB) cycle, which are products of highly transcribed genes under standard growth conditions, decreased dramatically after a 1-h heat shock at 47°C (Figure 2). Notably, *nblA* transcript levels increased ∼25-fold after heat shock, indicating that phycobiliprotein degradation is induced in a manner similar to the response to nutrient limitation in *Synechococcus* 7002 (Ludwig and Bryant, 2012). Furthermore, mRNA levels for genes encoding RNA polymerase, ribosomal proteins, and enzymes involved in heme and chlorophyll biosynthesis decreased. Genes involved in photoautotrophy were not the only ones subjected to down-regulation upon heat shock. Transcript levels for genes encoding the enzymes required for glycolysis (Embden–Meyerhof–Parnas pathway), the oxidative pentose phosphate cycle, pyruvate degradation, and the TCA (tricarboxylic acid) cycle (Zhang and Bryant, 2011) also decreased (Figure 2). These data impressively demonstrate that the metabolism of this cyanobacterium switches from normal cell growth (and the biosynthesis of the components required for growth) to metabolism for survival. This includes the biosynthesis of molecular chaperones to refold enzymes and structural components to maintain a minimally functional metabolic state. The results from this study for a culture subjected to a 1-h heat shock at 47°C are in general agreement with a microarray-based study of the heat-shock response of *Synechocystis* sp. PCC 6803 after shifting the culture from 34 to 44°C for 1 h (Suzuki et al., 2005, 2006).

The *Synechococcus* 7002 genome encodes two flavoproteins (*flv1* and *flv2*), which are required for a coordinated electron transfer directly to O_2_ and to prevent the formation of reactive oxygen/nitrogen species (Helman et al., 2003; Hackenberg et al., 2009). Transcripts for *flv1* increased approximately fourfold upon heat shock, whereas *flv2* transcripts decreased approximately twofold (Figure 2). Furthermore, transcripts for *sodB*, encoding superoxide dismutase, increased approximately fourfold after heat shock, but *katG* (catalase) transcripts decreased approximately twofold. Although the *flv1* transcripts increased, the change was far less than in cells acclimated to long-term CO_2_ limitation and was rather in the range observed for other short-term nutrient limitations or changes in irradiance levels (Ludwig and Bryant, 2011, 2012). The change in *sodB* transcript level, however, was the highest observed for this gene under any condition; *sodB* transcripts otherwise only increased significantly in cells grown at high salinity (see below). These data suggest that heat shock increases oxidative stress and that cells can produce a short-term acclimative response to this stress.

### Oxidative stress response to methyl viologen treatment

As noted above, heat shock caused a limited response to oxidative stress. To investigate the effect of oxidative stress on the transcriptome of *Synechococcus* 7002, a culture was incubated with methyl viologen in light. Methyl viologen accepts electrons from the reduced Fe-S clusters of PS I and transfers them directly to O_2_ to produce superoxide (Fujii et al., 1990; Yu et al., 1993). Superoxide produces other reactive oxygen and nitrogen species (ROS/RNS) that are harmful to most cellular components (e.g., see Scott et al., 2010). To induce oxidative stress, cells that had been grown under standard conditions were further incubated in presence of 5 μM methyl viologen for 30 min. The transcriptome of this culture was nearly identical to that for cells grown under standard conditions (Figure 1D). The transcript levels of very few genes changed by more than twofold. In a comparable microarray-based study in *Synechocystis* sp. PCC 6803, only 11 genes exhibited significant changes when cells were incubated with methyl viologen and kept at the same irradiance (Kobayashi et al., 2004), but more than 600 genes were considered to be differentially transcribed when cells were treated with H_2_O_2_ (Li et al., 2004). The data generated here using deep-sequencing of cDNAs have higher resolution, and these studies show that the response to superoxide-induced oxidative stress in *Synechococcus* 7002 is extremely limited, even if one considers genes that have low transcript abundances. Remarkably, transcript levels for superoxide dismutase (*sodB*), catalase (*katG*), peroxidases (SYNPCC7002_A0117, SYNPCC7002_A0970), cyanoglobin (*glbN*), and methionine sulfoxide reductases (*msrA* and *msrB*), whose products are all involved in inactivating ROS/RNS or in repair of the damage caused by these agents, did not increase (Table S3 in Supplementary Material). This suggests that ROS/RNS do not act as alarmones for the expression of these genes. These observations are generally consistent with previous observations that genes involved in amelioration of oxidative stress are constitutively expressed at high levels in *Synechococcus* 7002 (Ludwig and Bryant, 2012). Other regulatory mechanisms must account for the fourfold increase of *sodB* transcripts observed following heat shock.

### Growth at different salinities: Acclimation to ionic strength

**Figure 3:**
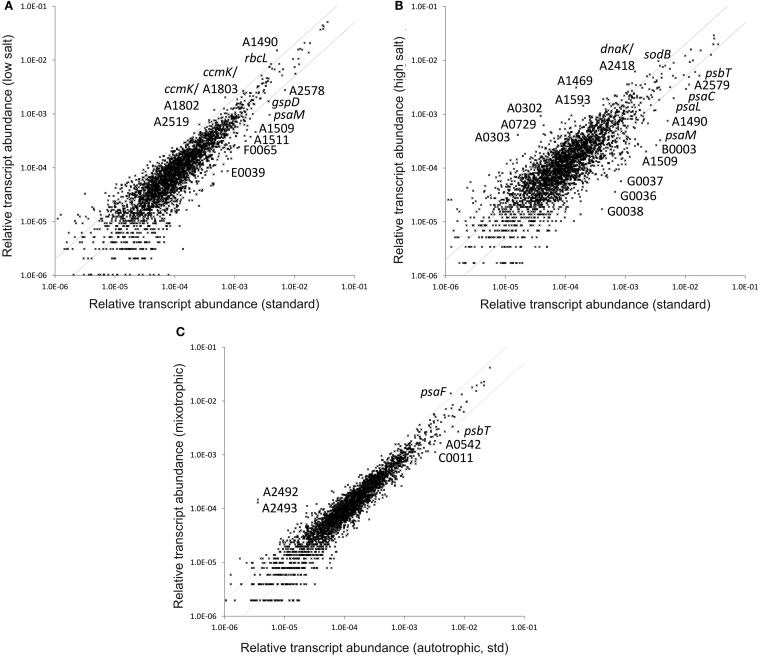
**Changes of the relative transcript abundance when cultures were grown at different salinities and in response to mixotrophic growth**. The scatter plots show the relative transcript abundances of cultures grown at **(A)** low salinity and **(B)** high salinity compared to growth in non-modified medium A^+^ under standard conditions. **(C)** Shows the scatter plot of the relative transcript abundance of a culture grown photomixotrophically with 10 mM glycerol as carbon source compared to photoautotrophic (standard) growth. The values for the standard conditions were calculated as the mean for three biological replicates. The gray lines give twofold changes in either direction. Selected genes are identified by name/locus tag number.

Because *Synechococcus* 7002 is a marine/euryhaline cyanobacterium, its growth medium is supplemented with salt (300 mM NaCl and 8 mM KCl). However, in its natural, estuarine habitat *Synechococcus* 7002 is probably subjected to frequent and large changes in salinity. Therefore, we compared the global transcriptomes of cells that were grown at different salinities. The transcriptome of cells grown at lower salinity (3 mM NaCl and 0.08 mM KCl), showed relatively minor changes compared to cells grown under standard conditions (300 mM NaCl and 8 mM KCl; Figure 3A), which is also reflected by the essentially unchanged growth parameters. When cells were grown at high salinity (1500 mM NaCl and 40 mM KCl), cells grew more slowly; the doubling time was almost three times longer than that of cultures grown under standard conditions (Table 1). The transcriptome of cells grown at high salinity differed significantly from that of cells grown under standard conditions (Figure 3B). Several of the changes were similar to observations that have been made in *Synechocystis* sp. PCC 6803 upon long-term high-salt acclimation (Marin et al., 2004).

Transcript levels for genes encoding the subunits of PS I decreased slightly in cells grown at high salinity (Figure 2). This differs from observations in *Synechocystis* sp. PCC 6803, for which it has been reported that transcript levels decrease for genes encoding subunits of both photosystems. Furthermore, the mRNA levels for most of those genes returned to the initial levels upon long-term acclimation to high salt (Marin et al., 2004). The transcript levels for genes encoding PS I subunits were at about standard levels in a culture grown at low salinity (Figure 2). In general, transcript levels for genes encoding PS II subunits did not change in response to salinity differences, but the situation with *psbA* paralogs was more complex. Compared to standard growth conditions, transcript levels for SYNPCC7002_A2164 and SYNPCC7002_A0157 increased fourfold and twofold, respectively, whereas transcripts for SYNPCC7002_A1418 decreased ∼1.5-fold in cells grown at 1.5 M NaCl (Table S3 in Supplementary Material). Furthermore, mRNA levels for genes encoding phycobiliproteins and enzymes required for their maturation were largely unchanged in cells grown at higher or lower salinity (Figure 2). However, *nblA* transcripts increased slightly in cells grown at 1.5 M NaCl, which suggested that cells reduce their phycobiliprotein content slightly at high salinity.

Changes of mRNA levels in response to growth at higher salinity were also observed for subunits of the NADH dehydrogenase complex. Transcript levels for most genes increased by an average of about twofold (maximally 7.5-fold for *ndhJ*); however, transcript levels for several genes did not change and transcripts for *ndhE* decreased 1.5-fold (Figure 2; Table S3 in Supplementary Material). The *Synechococcus* 7002 genome encodes three *ndhD* genes (annotated as *ndhD*, *ndhD1*, and *ndhD2*) that are not components of cyanobacterial CO_2_ transporters. Interestingly, transcripts for *ndhD2* increased sixfold in cells grown with 1.5 M NaCl, but transcript levels for the other two *ndhD* paralogs did not increase at high salinity. Higher transcript levels for *ndhD2* have also been observed in *Synechococcus* 7002 cells that were subjected to different nutrient limitations and after a 1-h exposure to high irradiation. This led to the suggestion that this alternative subunit might modify the ratio of protons to electrons transferred through the NADH dehydrogenase complex (Ludwig and Bryant, 2011, 2012).

The *Synechococcus* 7002 genome includes two operons encoding cytochrome oxidases (denoted *cta-I* and *cta-II*, respectively). It has been shown that cytochrome oxidase-I serves as the principal terminal oxidase of the respiratory chain, whereas cytochrome oxidase-II was suggested to play a role as signal transducer to measure the redox balance and trigger oxidative stress responses (Nomura et al., 2006a,b). Transcript levels of the *cta-I* genes increased ∼2.5-fold and those of *cta-II* genes increased four to fivefold in cells grown at high salinity (Figure 2). These observations suggest that cells may increase respiration activity (as a result from increased carbohydrate degradation, see below) and/or have an increased need to eliminate excess reducing equivalents when cells are grown at higher salinity. The latter explanation is further supported by the observed threefold increase of *flv1* transcripts, which encodes one of the two flavoproteins, which transfer electrons to O_2_ (Figure 2). Interestingly, transcript levels for *sodB* and *katG* increased ∼3- and 1.5-fold, respectively, at higher salinity, but no changes were observed when oxidative stress was imposed by methyl viologen treatment in the light (see above). Transcripts for both cytochrome oxidase operons decreased slightly in cells grown at low salinity (Figure 2). Transcript increases for *cta-II* of similar magnitude to those observed here in response to higher salinity were also observed for cells that had been subjected to limitation for several nutrients (Ludwig and Bryant, 2012). These data collectively suggest that cells exposed to higher ionic strength probably become overly reduced and acclimate by initiating changes to eliminate excess reducing equivalents.

Genes encoding alternative components of the photosynthetic electron transport chain showed strongly increased mRNA levels in cells grown at higher salinity (Table S3 in Supplementary Material). Transcripts for *petC-II*, encoding a Rieske Fe-S protein paralog, increased 14-fold, the largest increase for this gene observed under any of the conditions tested in this study or in previous transcription profiling studies (Ludwig and Bryant, 2011, 2012). The transcript level for its paralog *petC*, however, was also slightly increased in a culture grown at high salinity. Transcript levels for *cytM*, which encodes a membrane-bound *c*-type cytochrome (cytochrome *c*_M_; Shuvalov et al., 2001), increased ∼12-fold in cells grown at high salinity. Increases of *cytM* transcripts in *Synechococcus* 7002 have also been reported for cells subjected to nutrient limitation or subjected to high irradiation for 1 h (Ludwig and Bryant, 2011, 2012). In *Synechocystis* sp. PCC 6803 *cytM* transcripts increased in response to low-temperature and high-light stress (Malakhov et al., 1999). Transcripts for *cytM* in *Synechococcus* 7002 also increased approximately three- to fourfold at 30 and 22°C, respectively (Table S3 in Supplementary Material).

The *Synechococcus* 7002 genome encodes four genes annotated as high-light-inducible proteins: SYNPCC7002_A0186, SYNPCC7002_A0602, SYNPCC7002_A0858, and SYNPCC7002_A1476. Transcript levels for these proteins are known to increase in response to high irradiation levels and under nutrient limitation (He et al., 2001; Kilian et al., 2008). These proteins may bind chlorophyll(s) and/or carotenoid(s), and they may play a role in binding these molecules when cells are acclimating to stresses to facilitate changes in the composition of the photosynthetic apparatus (Xu et al., 2004; Storm et al., 2008). Transcript levels for three of these genes increased 3- to 22-fold when cells were grown at high salinity; however, transcripts for SYNPCC7002_A0858 were unaffected (Table S3 Supplementary Material,). We previously reported that transcripts levels for these genes increased after a 1-h exposure to high irradiance and in response to limitation for several nutrients (Ludwig and Bryant, 2011, 2012). Compared to standard conditions, transcript levels for these four genes were essentially unchanged in cells grown at lower salinity; however, transcript levels increased 5- to 40-fold following a 1-h heat shock (Table S3 in Supplementary Material).

Transcript levels for genes encoding enzymes of the CBB cycle, the inducible CCM and bicarbonate transporters all increased in cells grown at higher salinity. Surprisingly, transcripts encoding enzymes of glycolysis (Embden–Meyerhof–Parnas pathway), the oxidative pentose phosphate cycle, and pyruvate oxidation also increased. Furthermore, transcripts for *ldhA* (D-lactate dehydrogenase) were about sixfold higher in cells grown at high salinity. It should be noted that transcripts for *ldhA*, which encode the enzyme that produces the major fermentation product of *Synechococcus* 7002, were largely unchanged in cells grown under dark oxic or anoxic conditions (Ludwig and Bryant, 2011). When a culture was grown at low salinity, transcript levels of genes encoding the CCM, bicarbonate transporters, and RuBisCO showed slight increases (1.5- to 3-fold; Figure 2). Transcript levels for other enzymes of the CBB cycle, however, did not change (Table S3 in Supplementary Material).

Slightly increased transcript levels were observed for the F_0_F_1_-type ATPase I (∼1.5-fold in average) in cells of a culture grown at high salinity (Figure 2). Interestingly, mRNA levels for genes encoding a putative Na^+^-transporting ATPase (N-ATPase; Dibrova et al., 2010) did not increase when cells were grown at high salinity. The mRNA levels for all of those genes decreased to some extent; however, the *p*-values for the respective comparisons were too variable to support any definitive conclusion concerning the N-ATPase-related genes (see Table S2 in Supplementary Material). This finding is nevertheless contradictory to the proposed role of this enzyme in Na^+^ transport. However, transcription patterns do not necessarily coincide with protein expression patterns (Fulda et al., 2006), and the cellular localization and amount of the N-ATPase is currently unknown.

The major response of cyanobacteria to higher ionic strength is the production of compatible solutes (glycosylglycerol and sucrose in the case of *Synechocystis* sp. PCC 6803) to increase the osmotic potential within the cell without the corresponding toxicity effects of high Na^+^ levels (Klähn and Hagemann, 2011). In *Synechococcus* 7002 glycosylglycerol/glucosylglycerate biosynthesis is catalyzed by glucosylglycerol-phosphate synthase (*ggpS*), glucosylglycerol 3-phosphatase (*stpA*), and glycerol-3-phosphate dehydrogenase (*glpD*). Transcripts for these genes increased up to fourfold in cells grown at high salinity compared to standard conditions (Figure 2). Smaller increases in transcripts (up to twofold) were also observed for genes encoding an osmolyte ABC-transporter (SYNPCC7002_A2036 to SYNPCC7002_A2039); these *Synechococcus* 7002 genes have highest similarity to *ggtABC* from *Synechocystis* sp. PCC 6803 (Marin et al., 2004). Interestingly, transcripts for genes encoding subunits of Na^+^/H^+^ antiporters increased up to 20-fold in *Synechococcus* 7002 cells grown at 1.5 M NaCl (Figure 2; Table S3 in Supplementary Material). These findings differ from observations that have been made for *Synechocystis* sp. PCC 6803 cells that have been acclimated for long periods to high-salt conditions. It was reported that many genes were transiently induced when 684 mM NaCl was added to freshwater medium BG 11, but only a few genes had higher mRNA levels after longer acclimation periods (Marin et al., 2004). Transcripts for SYNPCC7002_A1446 increased the most (∼30-fold) in *Synechococcus* 7002 cells grown at high salinity (Table S3 in Supplementary Material). This ORF is annotated as a glycosyltransferase, but its closest homolog (sll1723) in *Synechocystis* sp. PCC 6803 is not listed among those genes for which transcripts increased after long-term acclimation to high salinity (Marin et al., 2004). Transcript levels for this gene are very low in cultures grown under standard conditions as well as most other conditions (Table S1 in Supplementary Material). Detection of this gene may only have been possible using cDNA sequencing; it may have escaped detection in previous microarray studies because of its low transcript abundance.

Growth in a medium of low salinity caused the opposite effect on the transcript levels of genes encoding the osmolyte ABC-transporter: the mRNA levels decreased significantly (3- to nearly 100-fold; Figure 2; Table S3 in Supplementary Material). Transcripts for the genes involved in the synthesis of glycosylglycerol (*ggpS*, *glpD*, and *stpA*; four- to sevenfold lower) also decreased but to a lesser extent (Figure 2). Transcript levels for genes encoding subunits of Na^+^/H^+^ antiporters did not show a consistent response to low-salinity conditions. Although transcripts for *mnhC* increased 10-fold, transcript levels for most of the genes increased up to twofold and decreased by no more than threefold (Table S3 Supplementary Material,).

Transcripts for two genes, SYNPCC7002_A0302 and SYNPCC7002_A0303, increased ∼25-fold in cells grown at high salinity compared to cells grown under standard conditions (Figure 3B). This was the largest increase observed for these two genes for any condition that we have studied to date (this study; Ludwig and Bryant, 2011, 2012). The SYNPCC7002_A0302 product is predicted to have a ferritin-like domain, and a domain analysis showed that it contains a DPS domain (DNA protecting protein under starved conditions). DPS proteins mediate responses to oxidative and peroxidative stress through their ferritin-like activity, which protects DNA and other cellular macromolecules against oxidative damage by neutralizing the toxic effects of Fe^2+^ and H_2_O_2_ that form hydroxyl radicals from the Fenton reaction (Fe^2+^ + H_2_O_2_ → Fe^3+^ + OH^−^ + OH**^·^**; Chiancone and Ceci, 2010). SYNPCC7002_A0302 is similar to slr1894 of *Synechocystis* sp. PCC 6803. Transcript levels for slr1894 increased about 2.5-fold after long-term acclimation to high salinity (Marin et al., 2004). SYNPCC7002_A0303 (annotated as hypothetical protein) is predicted to have a ChaB superfamily domain; ChaB is a putative regulator of ChaA, which is a Na^+^/H^+^ (Ca^2+^/H^+^) antiporter in *E. coli* (Ivey et al., 1993; Osborne et al., 2004). In cells grown at low salinity the transcript levels for these two genes showed the opposite response: i.e., both decreased significantly. SYNPCC7002_A0302 decreased ∼30-fold and SYNPCC7002_A0303 decreased approximately fourfold. These data collectively suggest that SYNPCC7002_A0302 and SYNPCC7002_A0303 are somehow involved in triggering a response to high salinity by regulating sodium exporters and protecting against oxidative damage.

Transcript levels of one gene (SYNPCC7002_A1490) showed an inverse regulation pattern compared to SYNPCC7002_A0302 and SYNPCC7002_A0303. Transcript levels increased in cells grown at low salinity and decreased in cells grown under high salinity. The relatively abundant transcripts for SYNPCC7002_A1490 under standard conditions increased threefold further when cells were grown at lower salinity (Figure 3A), whereas transcript levels were about approximately sevenfold lower in cells grown at high salinity. SYNPCC7002_A1490 is annotated as a hypothetical protein with a signal peptide, and it has limited sequence similarity to some proteins annotated as outer membrane beta-barrel proteins and hemagglutinins.

It has been reported in a study in *Synechocystis* sp. PCC 6803 that many genes showed regulation at the transcription level upon a salt shock (addition of NaCl to freshwater BG11 medium to a final concentration of 684 mM); however, most of the observed changes were only transient, and most transcript levels either returned rather quickly (up to 2 h) or over the course of about 24 h to the initial levels (Marin et al., 2004). The data presented here clearly show that long-term acclimation to high salinity differs in *Synechococcus* 7002. For example, transcripts for genes encoding high-light-inducible proteins increased at high salinity and remained higher than in cells grown under standard conditions. Furthermore, transcripts for genes encoding enzymes of central carbohydrate metabolism were higher when *Synechococcus* 7002 cells were grown at elevated salinity, whereas related changes in *Synechocystis* sp. PCC 6803 were only transient (Marin et al., 2004). These differences probably reflect the fact that *Synechocystis* sp. PCC 6803 is a freshwater strain, whereas *Synechococcus* 7002 is a euryhaline/coastal marine cyanobacterium.

### Transcriptome of *Synechococcus* 7002 cells grown under mixotrophic conditions

The transcriptome of glycerol-adapted *Synechococcus* 7002 cells, which were grown mixotrophically in A^+^ medium supplemented with 10 mM glycerol under otherwise standard conditions, closely resembled that obtained for photolithoautotrophically grown cells (Figure 3C). Only a few genes showed more than twofold changes in either direction (indicated by the gray lines in Figure 3C). Interestingly, transcript levels increased ∼40-fold for two adjacent ORFs, SYNPCC7002_A2492 and SYNPCC7002_A2493, in glycerol-supplemented cells. Transcript levels for these two genes increased even more, up to ∼700-fold, in cells grown with limiting CO_2_. Smaller increases in transcript levels for these two genes occurred when cells were grown with a reduced nitrogen source or under high irradiance (Ludwig and Bryant, 2011, 2012). These observations suggest that the products of these two genes may be important when there are excess reducing equivalents in cells.

Almost no changes were observed for transcripts of the genes encoding the photosynthetic electron transport chain, including both photosystems, and the phycobiliproteins. This suggests that supplementing the growth medium with a reduced carbon source, glycerol, did not reduce the production of the photosynthetic apparatus at the transcriptional level (Figure 2). However, *nblA* transcripts increased slightly, approximately threefold, which might cause a slight reduction in the levels of phycobiliproteins in cells (Karradt et al., 2008). Transcript levels for RuBisCO and the carboxysomal carbonic anhydrase (*icfA*) increased very slightly (∼1.5-fold), but transcript levels for genes encoding the structural components of the carboxysome and other enzymes of the CBB cycle were similar to those in photolithoautotrophically grown cells under standard conditions (Figure 2). Transcript levels for the genes encoding the inducible CO_2_ uptake complex (*ndhD3*, *ndhF3*, *cupA*, and *cupS*), which is a variant form of the NADH dehydrogenase complex that traps CO_2_ as bicarbonate in the cell (Battchikova et al., 2011), and *sbtA*, encoding the sodium-dependent bicarbonate transporter, increased when cells were grown mixotrophically (two- to threefold). Transcripts of these genes have been demonstrated to increase more strongly when cells were grown under CO_2_ limitation and slightly when cells were grown at high irradiance (Ludwig and Bryant, 2011, 2012). These results suggest that providing *Synechococcus* 7002 with a reduced carbon source results in excess reducing equivalents, which cells attempt to remedy by adding additional capacity for CO_2_ fixation. This is similar to results which have been obtained in a previous study upon a 1-h high-light treatment of *Synechococcus* 7002 after cells were grown under standard conditions (Ludwig and Bryant, 2011). Growth experiments showed that cells grown in a medium supplemented with glycerol grew about 15% faster than cells grown in the standard A^+^ medium. This observation supports the suggestion that more reducing equivalents are channeled into CO_2_ fixation and subsequent cell growth. The assumption that cells have excess reducing equivalents when supplemented with a reduced carbon source is further supported by the observation that transcripts for the *flv1* and *flv2* flavoproteins, which catalyze a direct electron transfer to O_2_ to avoid ROS/RNS formation (Helman et al., 2003; Hackenberg et al., 2009), increased about 1.5- to 2-fold compared to levels in cells grown under standard photolithoautotrophic conditions (Figure 2).

Transcript levels for genes encoding the F_0_F_1_-type ATPase increased slightly in cells grown mixotrophically, whereas mRNA levels for genes encoding the putative Na-ATPase were nearly twofold lower than in cells grown lithoautotrophically. Transcripts for genes encoding subunits of the NADH dehydrogenase complex also increased slightly (Table S3 in Supplementary Material). As found for cells grown under high salinity (see above) or nutrient limitation (Ludwig and Bryant, 2012), transcripts for *ndhD2* exhibited a greater increase, about 2.5-fold. Collectively, the transcriptome data obtained for cells grown mixotrophically with glycerol showed that CO_2_ fixation is *not* lowered, but just the opposite occurred. Cells exhibited an apparently greater need to dispose of reducing equivalents, as reflected by higher transcript levels for genes encoding components required for *C*_i_ uptake and concentration, CO_2_ fixation, and oxygen reduction by flavoproteins.

## Conclusion

Global transcription profiling of *Synechococcus* 7002 showed relatively minor differences when cultures were grown at temperatures below the optimum (38°C). Genes for fatty acid desaturases, which change the desaturation level of membrane lipids and therefore modify membrane fluidity in response to decreased temperature (Aguilar and De Mendoza, 2006), were among those genes showing the greatest increases. Exceeding the optimum growth temperature for a limited time induces the heat-shock response and completely changes the global transcription pattern; these changes were the most severe observed for any growth conditions and/or additional treatments that we have investigated in this organism (this study; Ludwig and Bryant, 2011, 2012). All major metabolic pathways were strongly down-regulated at the transcription level, whereas transcripts for genes encoding chaperones increased sharply – by nearly 1000-fold for *hspA* encoding a small heat-shock protein.

In cells grown at high salinity, transcripts for genes encoding enzymes for compatible solute biosynthesis increased, as reported for other cyanobacteria (Marin et al., 2004; Klähn and Hagemann, 2011). Moreover, genes encoding some enzymes involved in electron transport to oxygen and in detoxification of ROS/RNS were more highly expressed. Two adjacent genes, SYNPCC7002_A0302 and SYNPCC7002_A0303, which are likely involved in regulating Na^+^ exporters and protecting against oxidative damage, had much higher transcript levels at high salinity. Conversely, transcript levels for these genes were much lower at low salinity. Otherwise changes were relatively minor at low salinity relative to the standard. When cells were grown mixotrophically with glycerol, the transcriptome resembled that of a CO_2_-limited culture: mRNA levels of genes involved in CO_2_ uptake and fixation increased as well as genes encoding flavoproteins that transfer excess reducing equivalents directly to O_2_. Because SYNPCC7002_A2492 and SYNPCC7002_A2493 transcripts dramatically increased both under mixotrophic conditions and CO_2_ limitation (Ludwig and Bryant, 2012), their products may play an important but previously unappreciated role in *C*_i_ acquisition.

## Conflict of Interest Statement

The authors declare that the research was conducted in the absence of any commercial or financial relationships that could be construed as a potential conflict of interest.

## Supplementary Material

The Supplementary Material for this article can be found online at http://www.frontiersin.org/Microbial_Physiology_and_Metabolism/10.3389/fmicb.2012.00354/abstract

Supplementary Table S1 **Transcript levels for all protein-coding genes and for all samples presented in this study, and the relative transcript abundances of the standard samples of a previous study (Ludwig and Bryant, 2011)**.

Supplementary Table S2 **Comparisons of the relative transcript levels for all protein-coding genes and all conditions**.

Supplementary Table S3 **Changes in the transcript level for genes of selected metabolic pathways**. The ratios of the relative transcript abundance of cultures grown at 22 and 30°C (instead of 38°C), after a 1-h heat shock at 47°C, after induction of oxidative stress (30 min incubation with 5 μM methyl viologen in light), of cultures grown in modified medium A^+^ at lower or higher salinity and of a mixotrophically grown culture that was supplemented with 10 mM glycerol compared to “standard” conditions are given. The samples 22 and 30°C, oxidative stress, and glycerol were generated using SOLiD 3 chemistry and were compared to the mean of three standard data sets that were generated with SOLiD 3. Samples heat shock, low salt, and high salt were generated using SOLiD 4 chemistry. These are compared to the mean of three standard data sets generated using SOLiD 4 chemistry. Genes listed in more than one biochemical pathway are indicated by *, #, $, and x, respectively. std, standard; S4, SOLiD 4 chemistry.
